# Three-Dimensional Lower-Limb Kinematics from Accelerometers and Gyroscopes with Simple and Minimal Functional Calibration Tasks: Validation on Asymptomatic Participants

**DOI:** 10.3390/s22155657

**Published:** 2022-07-28

**Authors:** Lena Carcreff, Gabriel Payen, Gautier Grouvel, Fabien Massé, Stéphane Armand

**Affiliations:** 1 Kinesiology Laboratory, Geneva University Hospitals, University of Geneva, 1205 Geneva, Switzerland; gautier.grouvel@unige.ch (G.G.); stephane.armand@hcuge.ch (S.A.); 2 Nantes Université, Movement-Interactions-Performance, MIP, UR4334, F-44000 Nantes, France; 3 Gait Up SA, 1020 Renens, Switzerland; gabriel.payen@gaitup.com (G.P.); fabien.masse@gaitup.com (F.M.)

**Keywords:** inertial measurement units, gait kinematics, lower limbs, tridimensional kinematics, clinical gait analysis

## Abstract

The use of inertial measurement units (IMUs) to compute gait outputs, such as the 3D lower-limb kinematics is of huge potential, but no consensus on the procedures and algorithms exists. This study aimed at evaluating the validity of a 7-IMUs system against the optoelectronic system. Ten asymptomatic subjects were included. They wore IMUs on their feet, shanks, thighs and pelvis. The IMUs were embedded in clusters with reflective markers. Reference kinematics was computed from anatomical markers. Gait kinematics was obtained from accelerometer and gyroscope data after sensor orientation estimation and sensor-to-segment (S2S) calibration steps. The S2S calibration steps were also applied to the cluster data. IMU-based and cluster-based kinematics were compared to the reference through root mean square errors (RMSEs), centered RMSEs (after mean removal), correlation coefficients (CCs) and differences in amplitude. The mean RMSE and centered RMSE were, respectively, 7.5° and 4.0° for IMU-kinematics, and 7.9° and 3.8° for cluster-kinematics. Very good CCs were found in the sagittal plane for both IMUs and cluster-based kinematics at the hip, knee and ankle levels (CCs > 0.85). The overall mean amplitude difference was about 7°. These results reflected good accordance in our system with the reference, especially in the sagittal plane, but the presence of offsets requires caution for clinical use.

## 1. Introduction

The use of wearable sensors, such as inertial sensors, for ecological and autonomous gait analysis arouses important interest in the scientific community. Their low cost, small size, ease of use and increased performance in terms of battery life and memory are, indeed, highly suitable for gait monitoring outside of conventional cutting-edge laboratories. This technology accessibility could particularly benefit patients who currently have no easy access to conventional 3D gait analysis.

Accelerometers and gyroscopes are the most commonly used inertial measurement units (IMU) in human motion analysis and physical activity monitoring [1]. They, respectively, sense linear acceleration along one or several axes and angular velocity about one or several axes. The difficulties in using IMUs to compute orientation are, first, the poor estimations in terms of accuracy or robustness due to various sources of error [2] and, second, the fact that they sense data in their local frame. The first difficulty refers to the inherent property of each type of sensor. Accelerometers are, indeed, not suitable to estimate orientation during dynamic tasks since they measure the gravitational acceleration in addition to actual accelerations due to movement. In addition, gyroscopes are not suitable for orientation estimation over a long time period since they contain noise and bias that cause cumulative error when the signal is integrated [3]. Magnetometers, which sense the magnetic field, are often combined with IMUs in order to estimate the yaw angle (heading). However, magnetometer sensors also suffer from inaccuracies due to field distortion in the presence of ferrous materials during the measurement [2], i.e., in every environment surrounded by electrical devices and, implicitly, in all gait laboratories. Sensor fusion algorithms (SFA) have, therefore, been developed to achieve the best orientation estimation, taking advantage of a smart sensor combination and leaving aside the less accurate information along the measure. To overcome the second issue and estimate the segment frames from the local sensor frames, sensor-to-segment (S2S) calibration steps are necessary. Numerous calibration methods have been proposed, as shown by two recently published reviews, including 54 and 112 articles, describing S2S methods for motion analysis [4,5]. Both reviews concluded that the studies cannot be compared as they all use different methods of calibration. Thus, in the absence of consensus regarding SFA and S2S calibration, each method using IMU for kinematics computation needs to be cautiously detailed and validated before its use in clinical applications.

Numerous studies have investigated the validity of inertial sensors to compute lower-limb kinematics. Two recent systematic reviews reviewed 39 and 14 studies reporting validity metrics on 3D kinematics during simple (movements performed only in one plane of movement) and complex movement, such as walking [6,7]. The root mean square errors (RMSE) and correlation coefficients (CC) were shown to vary greatly across the studies (hip: RMSE [0.2–9.3]°, CC [0.53–1.00]; knee: RMSE [0.7–11.5]°, CC [0.4–1.00]; ankle: RMSE [0.4–18.8]°, CC [0.33–0.99], in 3D [6,7]). The studies mostly agreed that kinematics generally demonstrated good validity in the sagittal and frontal planes but were limited in the transverse plane. These observations are often drawn from small and heterogeneous studies [7] and need to be further documented.

The aim of this study was to assess the concurrent validity of a new IMU-based 3D lower-limb kinematics computation method on a healthy population against the silver clinical standard: the optoelectronic system with markers on anatomical landmarks. The computation method was also tested on optoelectronic data from clusters of markers, in order to separate the SFA and S2S sources of errors.

To be incorporated into clinical practice, new IMU-based gait analysis systems must report in similar form to be accepted clinical concepts [8]. This is not the case for a large majority of existing studies, which focused solely on one joint or one plane. Furthermore, some systems rely on S2S calibration tasks (such as functional tasks [9] or pointing tasks [10]) for every single segment axis to determine which considerably increase the time needed and the difficulty to perform the whole measurement procedure. The proposed method was, thus, thought to be suitable for pathological subjects and clinical settings, keeping the number and the difficulty of the instructed tasks as low as possible, providing the same kinematic outputs as commonly provided by conventional gait analysis [11]. Thus, pelvis and foot kinematics was assessed in addition to hip, knee and ankle kinematics. Finally, our method relied only on accelerometers and gyroscope to avoid any supplementary difficulties for the experimenter to deal with, such as magnetic environment preparation.

## 2. Materials and Methods

### 2.1. Participants

Ten healthy and asymptomatic adults were recruited. They were included if they were willing to participate and if they provided informed consent. They were excluded if they had muscular or skeletal pain or any disease significantly influencing gait, if they underwent surgery in the last 12 months, if they were pregnant and if they had a known allergy to hypoallergenic adhesive tape. The protocol was approved by and carried out in accordance with the hospital’s institutional ethical committee.

The sample size calculation was performed using the means and standard deviations of the RMSE reported in two validation studies on healthy subjects [12,13] evaluating the 9 following outcomes: hip, knee and ankle angles in flexion/extension, abduction/adduction and internal/external rotations, between an IMU system and an optoelectronic reference system. The sample size was calculated for each outcome and each study using the G*Power software, fixing the power to 0.95 and α to 0.05. The maximal sample size computed, among all outcomes extracted from the 2 reference studies assessing healthy subjects, was 9. An extra subject was added for security (margin to assure the completion of each subgroup).

### 2.2. Equipment

**Figure 1:**
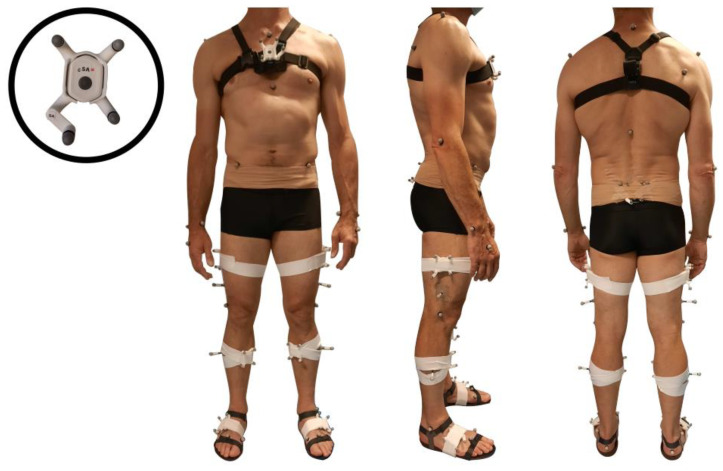
Equipment including 7 inertial sensors (Physilog6S, GaitUp) and reflective markers on the clusters and body landmarks (Conventional Gait Model 1.0).

Figure 1 shows the equipment set on all study participants. Participants were simultaneously measured by a 7-IMUs (Physilog6S, GaitUp, Renens, Switzerland) inertial system and a twelve-camera (Oqus7+) optoelectronic system (Qualisys, Göteborg, Sweden). The IMUs were inserted into 4-marker clusters (Figure 1) in order to track the position and orientation of the IMU in the global laboratory frame. The IMUs were positioned on the lower back (at sacrum level, in the middle and below the posterior superior iliac spines), the thighs (on the lateral side), the shanks (on the anterior and medial side) and the feet (on the top) (Figure 1). These locations were chosen as they are supposed to provide limited soft tissue artifacts [14]. Double-sided adhesive tape and elastic bands (SuperWrap straps, Qualisys, Göteborg, Sweden) were used to firmly fix the clusters and the IMUs on the participant’s body (Figure 1). The tridimensional acceleration and angular velocity were acquired at 256 Hz, with ranges of ±16 g and ±2000 °/s respectively. Reflective markers were placed according to the Conventional Gait Model (CGM) 1.0 [15] with the addition of two markers on the first metatarsal heads to help deal with any confusion between the ‘TOE’ marker and the cluster markers (Figure 1). Marker trajectories were measured at 100 Hz.

As shown in Figure 1, participants also wore pressure insoles (Moticon, Munich, Germany) fixed to light sandals and an IMU on the thorax fixed with a GoPro harness (GoPro, San Mateo, CA, USA). This was for the need of another project. Insoles and upper-body inertial data were not analyzed in this study. In addition, the high IMU sampling frequency was chosen to enable the acquisition of higher velocity tasks such as sprinting tasks, which were not used in the current study. The whole dataset including marker trajectories, IMU data, and insoles data is available online (Grouvel G. 2022. Human gait and other movements—markers/inertial sensors/pressure insoles/force plates; Yareta; https://doi.org/10.26037/yareta:kavwr4mzwzcjzepd6gp4cpkdz4).

### 2.3. Protocol

At the beginning and at the end of the measurement session, a trial involving the simultaneous acquisition of acceleration of one Physilog and the trajectory of one reflective marker, both fixed on a wand that felt down on the ground with a jerk movement was performed. These trials served as system synchronization trials and are referred to as *sync trials* in the rest of the paper.

**Figure 2:**
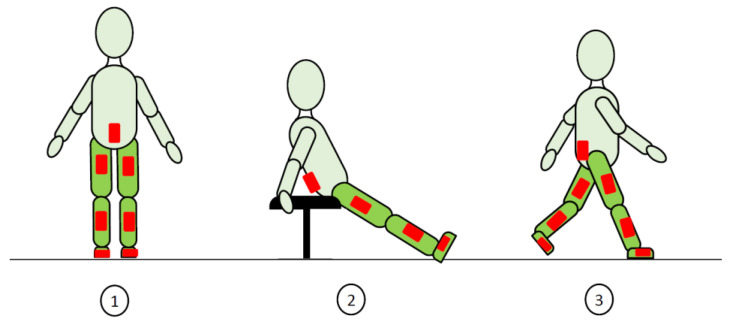
Tasks included in the protocol. (**1**) upright standing, (**2**) sitting with legs extended and (**3**) walking. IMUs are symbolized by the red boxes.

Each participant was asked to stand still in a neutral pose with the legs as vertical as possible and parallel feet, then to sit on a stool with the legs extended, pelvis inclined and toes off the ground (Figure 2), and finally to walk back and forth along the 10 m walkway at spontaneous speed. A minimum of 8 walking trials were assessed and analyzed per participant.

### 2.4. Data Processing

**Figure 3:**
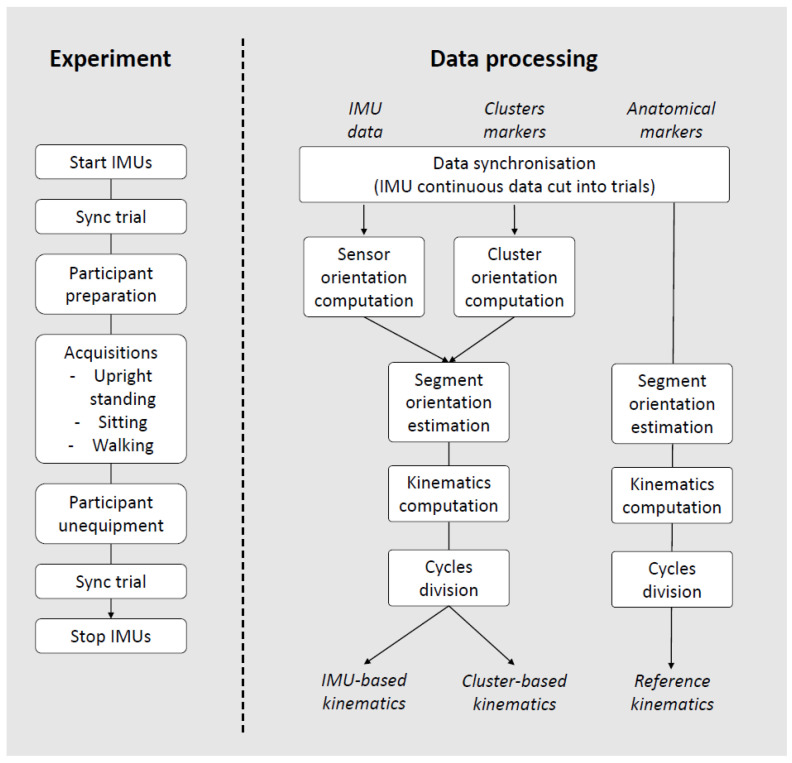
Flowchart of the experimental procedure and data processing main steps.

Data processing was performed using Matlab R2019 software (Mathworks, Natick, MA, USA). The main steps are presented in Figure 3.

#### 2.4.1. Pre-Processing

The absolute time of each Qualisys trial and the time of the impact captured during the *sync trials* were used to synchronize the optoelectronic and inertial systems and cut the inertial data into separated trials. The gaps in the marker trajectories were automatically filled using information of inter-correlated markers obtained from a principal component analysis [16]. The gait events were computed from the feet and pelvis marker trajectories as proposed by Zeni et al. [17].

#### 2.4.2. Sensor Orientations

Each cluster’s coordinate system was defined based on three markers and was converted into quaternions to obtain the cluster’s orientation varying in time. The sensor fusion proposed by Madgwick et al. was used to compute the sensors’ orientations (R_Pelvis sensor__→IF_^p^_,_ R_Thigh sensor__→IF_^t^_,_ R_Shank sensor__→IF_^s^_,_ R_Foot sensor__→IF_^f^, with IF^i^: initial frame of the sensor i) from accelerations and angular velocities [18], with the following fine-tuned parameters: sample period = raw IMU recording sample period, initial quaternion = rotation to gravity estimated with the five first accelerometer samples, and beta (algorithm gain) = 0.1.

#### 2.4.3. Sensor-to-Segment (S2S) Calibration

The following 3 steps for S2S calibration were proposed. Each of them consisted of determining a rotation between the actual sensor frame and the desired segment’s frame (R_j sensor__→j segment,_ with j: pelvis, thigh, shank or foot). The orientations computed from the clusters underwent the same rotations in order to check the relevance of the calibration performed on each sensor.

1. Alignment with gravity: During the first calibration standing posture, the segment’s vertical axis (Z) is supposed to be aligned with gravity measured by the accelerometer. A rotation was applied to the sensor data to align the vertical axis of the sensor with the vertical axis of the segment. This rotation was also applied to the cluster-based quaternions.
2. Alignment with the segment’s mediolateral (Y) axis: During the walking trials, the feet, shanks and thighs’ mediolateral axis was determined by the principal axis of the measured angular velocity, supposing that the movement occurs mainly in the sagittal plane for these segments. A rotation was applied to the corresponding sensor data to align the mediolateral axis of the sensor with the principal axis of movement during gait. This same rotation was applied to the cluster-based quaternions. The mediolateral axis of the pelvis was supposed to be manually aligned with the mediolateral axis of the sensor since no assumption could be made on the principal axis of movement during gait for this segment.
3. Mediolateral axis correction: The cross product of the 2 detected sensors’ vertical axes during standing and sitting postures allows one to know the direction of the mediolateral axis. The correction of the sign of the mediolateral axis previously determined was applied on the sensor and cluster-based orientations if necessary.

**Figure 4:**
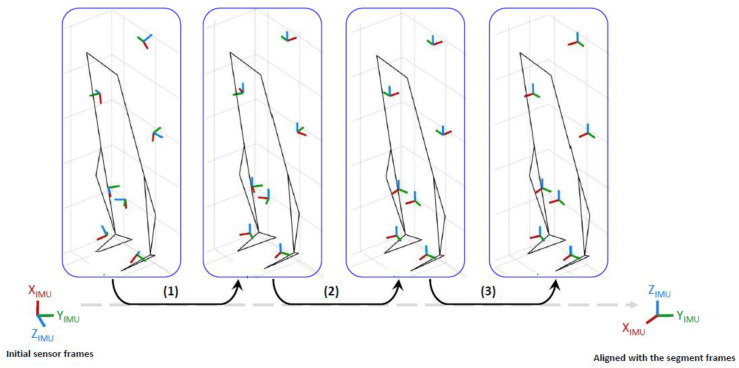
Illustrations of the S2S steps from the initial sensor frames to the segment frames in the standing pose. (**1**) Z alignment (blue axis) thanks to the gravity measured by the accelerometers during standing posture, (**2**) Y alignment (green axis) with the principal axis of movement measured by the gyroscopes during gait, (**3**) Correction of the Y axis direction thanks to the acceleration measured during the sitting posture leg extended.

Figure 4 illustrates the 3 first above-described S2S calibration steps on IMU and cluster data.

The desired segment’s orientations were, thus, determined by Equation (1):

R_Pelvis segment->IF_^p^ = R_Pelvis segment__→Pelvis sensor/cluster_ * R_Pelvis sensor/cluster__→IF_^p^,
R_Thigh segment->IF_^t^ = R_Thigh segment__→Thigh sensor/cluster_ * R_Thigh sensor/cluster__→IF_^t^,
R_Shank segment->IF_^s^ = R_Shank segment__→Shank sensor/cluster_ * R_Shank sensor/cluster__→IF_^s^,
R_Foot segment->IF_^f^ = R_Foot segment__→Foot sensor/cluster_ * R_Foot sensor/cluster__→IF_^f^, (1)

#### 2.4.4. Sensors Common Frame Setting

A common frame (CF) to all sensors (R_Pelvis segment__→CF,_ R_Thigh segment__→CF,_ R_Shank segment__→CF,_ R_Foot segment__→CF_) was set (only for IMUs, not applied to cluster data) (Equation (2)). During the standing posture, all sensors’ frames are forced to be aligned with the same azimuth axis (X).

R_Pelvis segment->CF_ = R_Pelvis segment->IF_^p^ * R_IF_^p^_→CF_,
R_Thigh segment->CF_ = R_Thigh segment->IF_^h^ * R_IF_^h^_→CF_,
R_Shank segment->CF_ = R_Shank segment->IF_^s^ * R_IF_^s^_→CF_,
R_Foot segment->CF_ = R_Foot segment->IF_^f^ * R_IF_^f^_→CF_, (2)

#### 2.4.5. Sensor-to-Global Calibration

The direction of travel was determined and the drift around the Z axis was corrected (only for IMUs, not applied to cluster data). During the stance phase of gait, the X axis of the segments was oriented along the direction of travel and corrected for each gait trial to set a global frame (GF) to all sensors (R_Pelvis segment__→GF,_ R_Thigh segment__→GF,_ R_Shank segment__→GF,_ R_Foot segment__→GF_) that aims to be aligned with the optoelectronic global frame. An additional step is used as a drift compensation since the common frame (CF) tended to drift over time (as represented by R _CF_^i^_drifted__→CF_ in Equation (3)).

R_Pelvis segment->GF_ = R_Pelvis segment->CF_^p^_drifted_ * R _CF_^p^_drifted__→CF_ * R_CF__→GF_,
R_Thigh segment->GF_ = R_Thigh segment->CF_^t^_drifted_ * R _CF_^t^_drifted__→CF_ * R_CF__→GF_,
R_Shank segment->GF_ = R_Shank segment->CF_^s^_drifted_ * R _CF_^s^_drifted__→CF_ * R_CF__→GF_,
R_Foot segment->GF_ = R_Foot segment->CF_^f^_drifted_ * R _CF_^f^_drifted__→CF_ * R_CF__→GF_, (3)

Steps (2) and (3) were unnecessary for the clusters as all the segment orientations were directly given with respect to the same optoelectronic global frame, instead of the relative inertial frame IF^i^ for the IMUs.

#### 2.4.6. Kinematics Computation and Cycle Division

From the determined segment frames, the joint kinematics were computed with Euler rotations following CGM 1.0 [15] (Equation (4)), and the reference kinematic data were computed using the same convention.

R_Hip_ = R_Pelvis segment__→Thigh segment_ = R_Pelvis segment__→GF_ * R^−1^_Thigh segment__→GF_,
R_Knee_ = R_Thigh segment__→Shank segment_ = R_Thigh segment__→GF_ * R^−1^_Shank segment__→GF_,
R_Ankle_ = R_Shank segment__→Foot segment_ = R_Shank segment__→GF_ * R^−1^_Foot segment__→GF_, (4)

The pelvis kinematics was computed directly from R_Pelvis segment->GF_ and the foot kinematics (used for the foot progression angle) was computed as follows: R_Foot_ = R_Foot segment__→Pelvis segment_ = R_Foot segment__→GF_ * R^−1^_Pelvis segment__→GF_.

It was estimated that our system synchronization showed an accuracy of about 1s. Such time drifts appeared between the kinematic curves obtained from the reference and the IMUs in the gait trial results and were corrected using a signal cross correlation. Resulting kinematic data were then cut into gait cycles, using the events detected on the raw inertial data as proposed by Mariani et al. [19] for the IMU data and using the events detected by the previously described method from marker trajectories [17] for the clusters.

### 2.5. Data Analysis

Eleven kinematic variables per side were compared between the 3 approaches (reference kinematics from the anatomical markers, kinematics from the clusters, and kinematics from the IMUs): pelvis ante/retroversion, pelvis obliquity, pelvis in/external rotations, hip flex/extension, hip ab/adduction, hip in/external rotations, knee flex/extension, knee ab/adduction, knee in/external rotations, ankle dorsi/plantar flexion and foot progression angle. These outcomes were selected as they are of interest for clinical gait analysis [20]. The foot progression angle was defined as the angle between the pelvis antero-posterior axis and the foot longitudinal axis. The kinematic data were represented according to the gait cycle (%) and averaged for each participant.

The RMSE, RMSE centered at the mean (as proposed in [21]), the Pearson’s correlation coefficient (CC) and the absolute difference in ranges of motion (ΔROM) were computed to evaluate, respectively, the global accuracy, the accuracy without any offset, the shape of the curves and the amplitudes of the curves. Altman’s guidelines were used to interpret the correlations: poor, if CC < 0.20; fair, if 0.20 ≤ CC < 0.40; moderate, if 0.40 ≤ CC < 0.60; good, if 0.60 ≤ CC < 0.80; and very good, if CC ≥ 0.80 [22].

## 3. Results

Among the 10 participants measured, one had a technical issue with the sensor located on his left thigh, so his left-side results are missing. Six men and four women, age: 30.2 ± 6.7 years, height: 173.5 ± 6.8 cm, and mass: 68.0 ± 14.6 kg, were included in the analysis. Ten to twelve gait trials were captured per participant.

**Figure 5:**
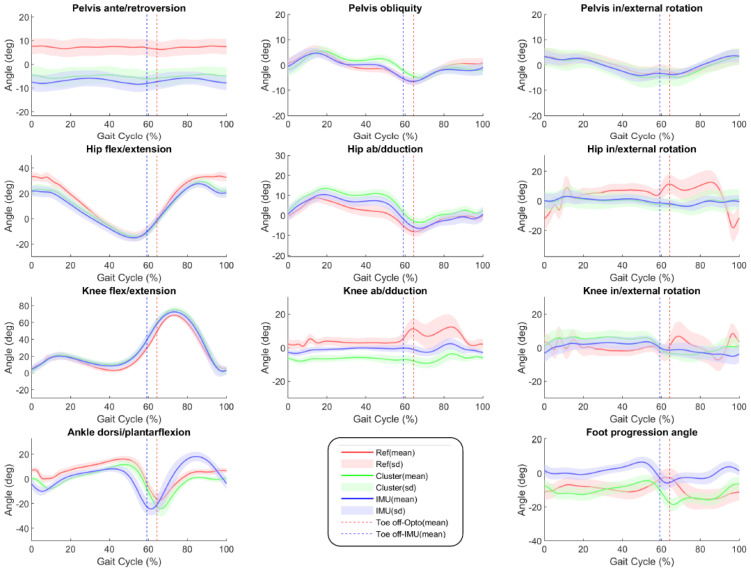
Mean kinematic curves (±standard deviation ‘sd’) of all participants’ right sides. In red: the reference (‘Ref’) kinematics from anatomical markers; in green: the kinematics obtained from the clusters; in blue: the kinematics obtained from the IMUs. The dashed lines correspond to the mean toe off events detected, in red: detected from the anatomical markers with the optoelectronic (‘Opto’) system; in blue: detected from the IMUs located on the feet.

**Figure 6:**
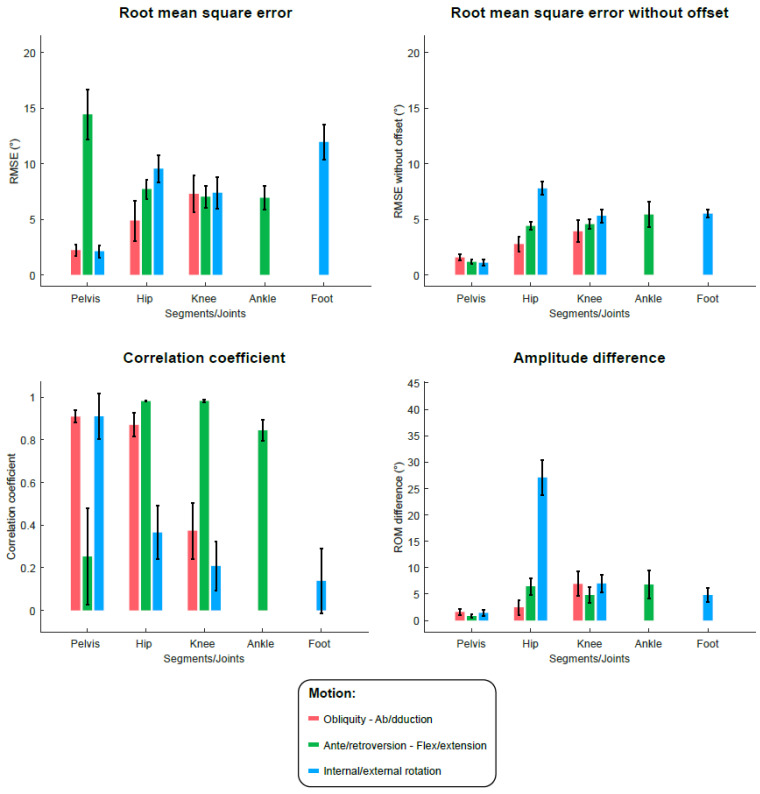
Metrics (root mean square error (RMSE), RMSE centered at the mean, correlation coefficient and difference in range of motion (ROM)) of validity evaluation of the method applied to the IMU data against the optoelectronic reference. Histograms represent the mean values (and interval confidence bars) for all participants, for each joint/segment and each plane.

**Figure 7:**
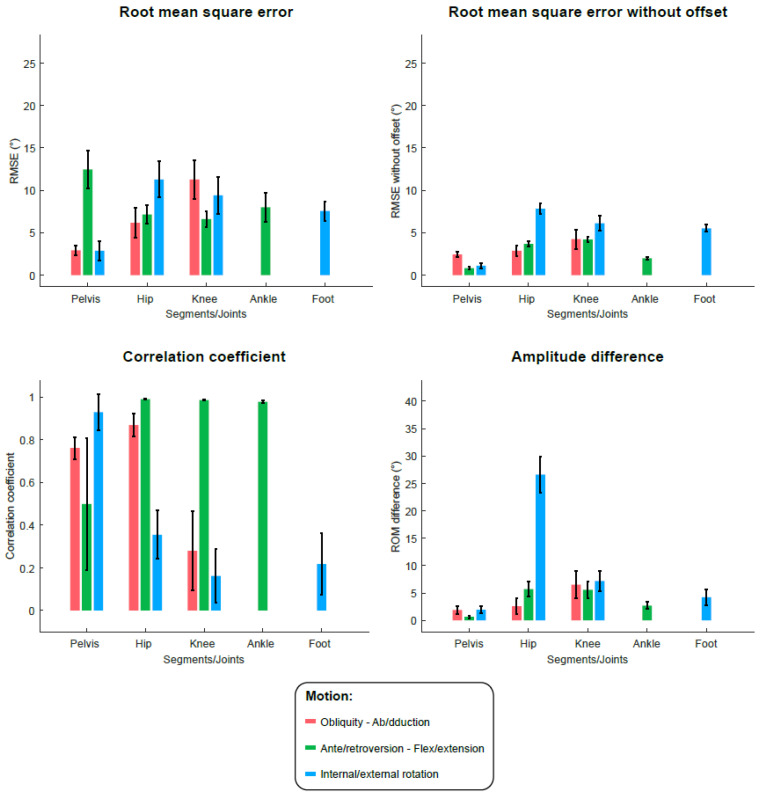
Metrics (root mean square error (RMSE), RMSE centered at the mean, correlation coefficient and difference in range of motion (ROM)) of validity evaluation of the method applied to the cluster data against the optoelectronic reference. Histograms represent the mean values (and interval confidence bars) for all participants, for each joint/segment and each plane.

Figure 5 illustrates the kinematic results from IMUs and clusters as mean curves and standard deviation areas for the whole participants. The RMSE, RMSE centered at the mean, CC and ΔROM results regarding the IMU and cluster-based kinematics are presented in Figure 6 and Figure 7, respectively, and the associated tables are available as Supplementary Material (Table S1). The overall mean RMSE is 7.5° for IMU-kinematics and 7.9° for cluster-kinematics. The maximal errors were found at the pelvis ante/retroversion level (RMSE = 14.1 ± 2.8° for IMUs and 12.1 ± 2.8° for clusters). After the offset removal, the overall mean centered RMSE was 4.0° for IMU-kinematics and 3.8° for cluster-kinematics. The highest errors were then found at the hip internal/external rotation level (centered RMSE = 7.8 ± 1.3° for IMUs and 7.8 ± 1.4° for clusters). Regarding the curve shape accordance, we found very good CCs in the sagittal plane for both IMUs and cluster-based kinematics at the hip, knee and ankle levels. In addition, the hip ab/adduction, pelvis int/external rotations and pelvis obliquity showed very good correlations. In the other planes, the IMU and cluster kinematics curve shapes showed poor to moderate correlations with the reference. The mean amplitude differences (ΔROM) were 6.8° for IMU-kinematics and 6.1° for cluster-kinematics. The maximal ROM difference concerned the hip internal/external rotations with ΔROM up to 27.2 ± 7.4° with IMUs and 25.9 ± 7.4° with clusters.

## 4. Discussion

This study aimed at assessing the validity of a 3D lower-limb kinematics computation method based on IMUs on an asymptomatic population. The main results suggested that our method was comparable to the optoelectronic system, chosen as silver clinical standard. It ensured good global accuracy and accordance for the shape and the amplitude of the curves, but some exceptions must be acknowledged. The overall mean RMSEs were below 10°, with the exception of pelvic ante/retroversion and foot progression angle. The errors came essentially from an offset between the reference kinematics and the IMU-based kinematics. The removal of this offset provided overall mean RMSEs below 8° for all joints and planes. The kinematics of the sagittal plane showed better results with small errors without offset (<6°), very high correlations for the hip, knee and ankle flexion/extensions (CC ≥ 0.85) and low amplitude differences (<7°).

Although a lot of studies have already been published regarding gait kinematics from IMUs, few studies evaluated the complete 3D kinematics of the entire lower limbs as it was proposed in the current study. Studies often assessed one joint [9,23,24] or one plane [25,26,27]. The errors found in our study appeared higher than in other existing published methods. Focusing on those who assessed 3D lower-limb kinematics, we can compare with the following works. Lebleu et al. found errors varying between approximately 1° and 4° for most joints, planes and functional calibration movements, similar to the ones proposed in the current study [28]. It is noteworthy that the authors included the pelvis kinematics, as we did, since it is commonly presented in clinical gait analysis reports. They found errors below 1.5 ± 1.8° for pelvis tilt, obliquity and rotation. Nazarahari et al. found errors below 8.1° for ankle and knee angles in 3D [29]. In these two examples, the reference kinematics is based on markers located on the IMU boxes or associated clusters, which is the case for a large part of the studies in the current literature. Thus, the resulting kinematics is different from the clinical reference using markers located on anatomical landmarks. Indeed, the kinematics from IMUs is logically closer to cluster-based kinematics than anatomical-marker-based kinematics due to the same soft tissue artefacts. This has actually been verified by Teufl et al. who found significant different errors between the two above-mentioned reference kinematics and his IMU method (errors below 2.4° against cluster-based reference kinematics and below 6° against anatomical markers-based kinematics) [30]. For those who used anatomical-marker-based kinematics, the errors are closer to the ones presented in our study. Tadano et al. found errors between 7.8° and 10.1° for hip, knee and ankle flexion/extensions, using a combination of functional calibrations and additional pictures to perform S2S alignment [31]. Cho et al. managed to obtain lower errors (<4.4°) for hip, knee and ankle 3D kinematics with the use of a magnetometer, in addition to accelerometer and gyroscope, but very little information is available regarding the S2S alignment [32].

Our method was, indeed, solely based on accelerometer and gyroscope data. This choice was made to reduce the uncertainty of orientation estimation caused by magnetic distortions [33] but represented a supplementary challenge. Omitting magnetometer data, which serves as a global heading (horizontal) reference, entails the lack of a global reference frame for our IMUs and the lack of drift diminution in the transversal plane [30]. We, thus, had to compensate for this absence with the estimation of a sensor common frame and a sensor-to-global calibration. In general, magnetometer-free IMU systems have been reported not only to be equivalent to IMU systems using magnetometers [34] but, in some cases, to outperform them [30,33]. However, if particular care is taken in the preparation of the magnetic environment, magnetometer-based algorithms perform slightly better [35]. Given our perspective to further use our system in clinical conditions and the huge advantages of omitting magnetometer in a user perspective, it was worth a try. In any case, magnetometer-free systems are described as relevant for capturing data for the short term [36].

The main issue with our method concerned the offset, i.e., the constant absolute angle between the IMU kinematics and the reference kinematics. This was especially visible on the pelvis ante/retroversion, with a mean absolute RMSE of 14.1 ± 2.8° and a mean centered RMSE (removing the average mean of each curve) of 1.2 ± 0.3°. Berner et al. related very similar errors and tried to compensate them taking advantage of optical data during calibration trials [37]. This adjustment permits one to considerably reduce the overall errors (<5°, except hip rotation). The problem with this proposition is the need for an external device, which compromises the use of the gait analysis system outside of standard laboratories. Pacher et al. proposed a calibration method for the pelvis based on a combination of functional movements and the use of an external IMU fixed to a device, the inclination of which coincides with the line between the superior iliac spines (method inspired from Picerno et al. [10]) [38]. This combined method had the merit of being independent of any optical system and allowed a reduction in the error but remained superior to 10°, so it may be improved.

The curve shapes resulting from our method showed good to very good agreement with the reference, with the exceptions of foot progression angle (CC = 0.13), hip rotation (CC = 0.36), knee ab/adduction (CC = 0.38) and rotation (CC = 0.19), as well as pelvis tilt (CC = 0.25). These findings were in line with other studies [37,39]. In the frontal and transverse planes, the movement amplitudes are indeed smaller, as compared to the sagittal plane, resulting in low signal-to-noise ratios [40], which may explain low CCs. Very good sagittal kinematics curve shapes agreement was also largely observed in the literature [6,7] and this is very satisfying, knowing that the angles in the sagittal plane are considered as primary gait drivers [37] and, thus, highly consulted during the process of clinical gait analysis.

The foot progression angle computed with our method had one of the highest errors and lowest correlations with the clinical reference. Figure 5 illustrates the poor concordance between the kinematic curves. This could be explained by a difference in the definitions of these angles. In the CGM, the foot progression angle is defined as the angle between the global coordinate system and the foot longitudinal axis [15]. However, this definition was difficult to follow in our method using IMUs, given the lack of a global coordinate system due to the absence of magnetometers. In fact, our global coordinate system was set aligned with the direction of travel at each gait cycle. The foot progression angle, defined as the angle between the foot longitudinal axis and the direction of travel, has been tested from magneto-inertial sensors and showed satisfying results (RMSE < 3°) [41,42], but in our case, this was not stable enough. We, thus, chose to define the foot progression as the angle between the pelvis anteroposterior axis and the foot longitudinal axis.

The comparison of IMU-based kinematics and cluster-based kinematics allowed us to have an idea of the part of SFA and S2S in kinematics computation. RMSEs, centered-RMSEs, CCs and ΔROMs were very close in both methods, which means that SFA was correctly implemented and provided accurate sensor orientations. The main differences were observed at the ankle and foot level, where the cluster-based kinematics seemed to have lower error and better shape accordance with the reference than the IMU-based kinematics. We can, thus, infer that this came from SFA inaccuracies. Similar errors were found in other studies at the ankle flexion/extension level, specifically at mid and terminal swing phases [43,44]. This could be due to the abrupt difference in dynamics of the foot between the stance and swing phases, as it is known that SFA highly depends on dynamics of motion [2]. The sampling rate was found to have a great effect on Madgwick’s algorithm performance in dynamic conditions [18]. To adapt the sampling rate could constitute an axis of improvement for the method. This may also be caused by inaccuracies coming from the sensors’ common frame setting and the sensor-to-global frame calibration. Indeed, these two calibration steps were only performed on IMU data and not on cluster data, since markers are already measured in the global frame. On one hand, if the participant had the feet slightly rotated during the standing posture, the initial and common orientation between the sensors may be incorrect. On the other hand, if the participant had the feet slightly rotated during mid stance as compared to the direction of travel, the global frame may also be incorrect. These aspects must be improved in future work.

The way of dealing with the error of orientation that accumulates over time (drift) was satisfying. We constrained the segment orientations to be aligned with the direction of travel while being in mid-stance phase and did not notice a growing drift in the kinematic results. This was permitted thanks to the short and straight gait trials acquired. The drift could not be compensated similarly for longer or curved measurements [45]. In addition, particular attention will have to be paid when using this system with pathological individuals whose body segments do not necessarily point toward the direction of travel in stance phase (e.g., individuals walking in crouch gait with internally rotated hips and in-turned feet [46]).

### 4.1. Study Limitations

The sensor gains and offsets were not changed as compared to the ones provided by the constructor. However, we demonstrated that the kinematics computed from IMUs and the kinematics computed from markers rigidly fixed to the IMU boxes were very similar. Next, as mentioned previously, one strategy to set an initial common frame for all the sensors was to define it while the participant was standing straight during the calibration posture. This could have been performed by aligning the sensors on a specific device to ensure true inter-alignment, such as proposed in various existing methods [37]. Another limitation is that our S2S calibration method relies on the good segments’ alignment of the participant during standing and sitting postures, and the true segments’ principal rotation along the mediolateral axis during walking. This may provide errors when assessing pathological populations. Then, we must acknowledge that the sample size was low as compared to similar concurrent validity studies. The addition of some participants as well as some pathological individuals could be beneficial for a future study not only in order to strengthen the current results but also to assess the clinical relevance of our system. Finally, we did not assess the reliability of our method due to the heaviness of such a protocol, which constitutes a relevant perspective as well.

### 4.2. Clinical Relevance

A new 7-IMU system composed of accelerometers, gyroscopes and associated SFA and S2S algorithms was proposed in this work. Far from being the first of its kind, our method found its novelty in providing complete 3D lower-limb kinematics, similar to conventional gait analysis outcomes, with no need for an external device or high expertise for data acquisition. The absolute errors in our system against the clinical reference were globally too high to be used for clinical interpretations. McGinley et al. postulated that absolute errors of a maximum of 5° were seen as clinically reasonable [47]. We did not reach this objective. We believe that these errors can be caused by the inherent discrepancies in kinematics definitions. The clinical conventions taken as reference here are based on joint anatomical axes, whereas the IMU-based kinematics was rather built on functional axes. In fact, the CGM was used as the reference in the current study since it is the most widely used and understood biomechanical model for clinical gait analysis [15,20]. However, other models exist [48,49] and could be considered to concur with IMU-based kinematics. As previously discussed, models using clusters instead of anatomical markers (such as the calibrated anatomical landmark technique ‘CAST’) may better agree with IMUs given the resulting equivalent soft tissue artefacts. In addition, models using functional methods to determine joint center locations [50] could also better match our IMU-based system given the chosen S2S functional calibrations. Kinematics from IMUs may be comparable between sessions for the same subject, as can be seen from the low inter-trial variabilities and the excellent reliability reported in numerous studies [6]. When the offset is removed from IMU-based kinematics, we moved closer from the objective of 5° of errors, at least in the sagittal plane. This offset determination is a key improvement needed for our system. Indeed, the simple S2S calibration proposed in the current study, based on only three simple tasks asked to the participant, was not sufficient. Calibration procedures must be undertaken very carefully. This can imply the necessity to use additional devices, such as a video camera, an instrumented goniometer or additional IMUs, in order to eliminate the offsets and reach higher accuracies.

In its current form, the algorithm permitted good estimation of the joint ROM as well as the waveform, especially in the sagittal plane. If the system is to be used in a clinical context, this information could be sufficient for some pathological cases, such as those with light gait deviations (e.g., toe walkers, stroke survivors, patients with Parkinson’s disease or multiple sclerosis). For instance, if the purpose of gait analysis is to confirm that an Achilles tendon lengthening has permitted an increase in ankle dorsiflexion motion, the system could be adapted. However, absolute angles and the description of the frontal and transverse planes are, for instance, essential when dealing with patients with cerebral palsy (with a high level of disability). As an example, clinicians who need to devise derotation osteotomy could not rely on our system. Be that as it may, IMU systems do not seem ready to substitute optoelectronic systems but could complement them.

## 5. Conclusions

A 7-IMU system, associated calibration tasks and algorithms were proposed to easily compute 3D lower-limb kinematics from accelerometer and gyroscope data. This study found that the resulting curve patterns were comparable to the clinical standard but the absolute errors remained relatively high for clinical use outside of laboratories.

## Data Availability

Data supporting reported results can be found through this identifier https://doi.org/10.26037/yareta:kavwr4mzwzcjzepd6gp4cpkdz4 (Grouvel G. 2022. Human gait and other movements—markers/inertial sensors/pressure insoles/force plates; Yareta).

## Supplementary Materials

The following supporting information can be downloaded at: https://www.mdpi.com/article/10.3390/s22155657/s1, Table S1: Validity results regarding IMU-based kinematics and cluster-based kinematics against the anatomical markers-based reference kinematics for each joint/segment and plane.
